# Plasma extracellular vesicles in people living with HIV and type 2 diabetes are related to microbial translocation and cardiovascular risk

**DOI:** 10.1038/s41598-021-01334-y

**Published:** 2021-11-09

**Authors:** Beate Vestad, Tuula A. Nyman, Malene Hove-Skovsgaard, Maria Stensland, Hedda Hoel, Anne-Marie Siebke Trøseid, Trude Aspelin, Hans Christian D. Aass, Maija Puhka, Johannes R. Hov, Susanne Dam Nielsen, Reidun Øvstebø, Marius Trøseid

**Affiliations:** 1 Research Institute of Internal Medicine, Oslo University Hospital Rikshospitalet, Postboks 4590, 0424 Oslo, Norway; 2 Institute of Clinical Medicine, University of Oslo, Oslo, Norway; 3 Norwegian Society for Extracellular Vesicles, NOR-EV, Oslo, Norway; 4 Department of Immunology, Institute of Clinical Medicine, University of Oslo and Oslo University Hospital Rikshospitalet, Oslo, Norway; 5 Department of Infectious Diseases, University Hospital of Copenhagen Rigshospitalet, Copenhagen, Denmark; 6 Medical Department, Lovisenberg Diaconal Hospital, Oslo, Norway; 7 The Blood Cell Research Group, Department of Medical Biochemistry, Oslo University Hospital, Ullevål, Oslo Norway; 8 Institute for Molecular Medicine Finland FIMM, EV and HiPrep Cores, University of Helsinki, Helsinki, Finland; 9 Division of Surgery, Inflammatory Medicine and Transplantation, Norwegian PSC Research Center and Section of Gastroenterology, Oslo University Hospital Rikshospitalet, Oslo, Norway; 10 Section of Clinical Immunology and Infectious Diseases, Oslo University Hospital Rikshospitalet, Oslo, Norway

**Keywords:** Chromatography, Chronic inflammation, HIV infections, Proteomics

## Abstract

HIV and type 2 diabetes (T2D) are both associated with gut microbiota alterations, low-grade endotoxemia and increased cardiovascular risk. We investigated the potential role of plasma extracellular vesicles (EVs) in relation to these processes. Plasma EVs were isolated by size exclusion chromatography in fasting individuals with HIV and T2D (n = 16), T2D only (n = 14), HIV only (n = 20) or healthy controls (n = 19), and characterized by transmission electron microscopy, western blot, nanoparticle tracking analysis and quantitative proteomics. The findings were compared to gut microbiota alterations, lipopolysaccharide levels and cardiovascular risk profile. Individuals with concomitant HIV and T2D had higher plasma EV concentration, which correlated closely with plasma lipopolysaccharides, triglycerides and Framingham score, but not with gut microbiota alterations. Proteomic analyses identified 558 human proteins, largely related to cardiometabolic disease genes and upstream regulation of inflammatory pathways, including IL-6 and IL-1β, as well as 30 bacterial proteins, mostly from lipopolysaccharide-producing Proteobacteria. Our study supports that EVs are related to microbial translocation processes in individuals with HIV and T2D. Their proteomic content suggests a contributing role in low-grade inflammation and cardiovascular risk development. The present approach for exploring gut-host crosstalk can potentially identify novel diagnostic biomarkers and therapeutic targets.

## Introduction

Human immunodeficiency virus (HIV) and type 2 diabetes (T2D) are chronic diseases associated with low-grade inflammation and increased risk of cardiovascular disease (CVD)^1,2^. An increasing body of evidence has linked functional alterations in the gut microbiota to several chronic diseases, including HIV^3–5^, T2D^6,7^ and increased cardiovascular risk^8,9^. However, a complete understanding of the microbe-host crosstalk mediating this risk is lacking.

A possible factor in this process is the translocation of microbial products, such as lipopolysaccharides (LPS, endotoxin) across an impaired gut mucosal membrane to the circulation, also denoted microbial translocation^10^ or endotoxemia. Increased plasma levels of LPS have been linked to cardiovascular risk in T2D^8,11^, as well as dyslipidemia, platelet activation and endothelial dysfunction in HIV-infected individuals^12,13^. While translocated LPS is typically described as single molecules or associated with lipoproteins, LPS can also be released as a membrane-associated molecule through extracellular vesicles (EVs)^14^.

Cell-derived EVs, including bacterial outer membrane vesicles (OMVs), have emerged as important modulators of intercellular communication and host-microbe crosstalk^15–17^, including in inflammatory disorders and cardiovascular disease^18–20^. Along with the increased recognition of the gut microbiota in human disease^21,22^, we aimed to explore EVs as potential mediators in the crosstalk between microbial products, host inflammation and cardiovascular risk. Specifically, we set out to characterize and study the proteomic content of plasma EVs from patients with HIV and T2D, and compare EV-related parameters to functional changes in the gut microbiota and cardiovascular risk profile. A secondary aim was to investigate whether EVs and their proteomic content may present information on microbial translocation processes beyond circulating LPS in these patients.

## Results

### Characterization of size exclusion chromatography isolates confirmed the presence of EVs

**Figure 1:**
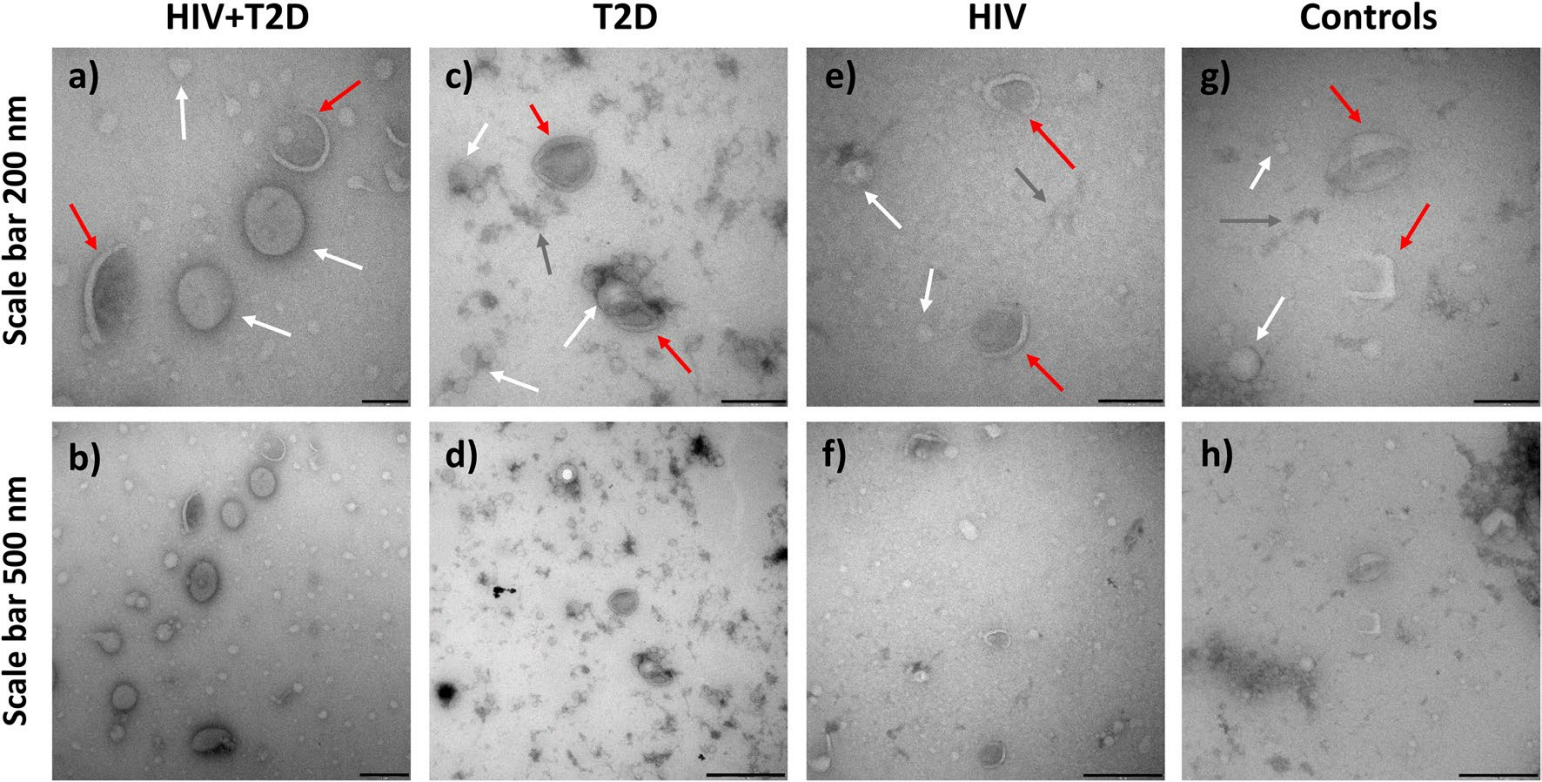
Transmission electron microscopy of SEC-isolated plasma EV pools from individuals with HIV + T2D (**a**, **b**), T2D (**c**, **d**), HIV (**e**, **f**) and controls (**g**, **h**). The top four close-up images have scale bars of 200 nm, whereas the bottom four show wide-field images of the same areas with scale bars of 500 nm. Different structures are highlighted with arrows, resembling EVs (red), lipoprotein particles (white) and protein aggregates or other matrix from plasma (dark grey).

The presence, morphology and purity of EV isolates were evaluated by transmission electron microscopy (TEM), displaying typical cup-shaped bilayer EV-like structures in pooled SEC isolates of the four study groups. Other molecular structures were also apparent in the preparations, which suggested the co-presence of plasma lipoprotein particles, identified by their non-collapsed very round shape and staining characteristics, and some other matrix, such as protein aggregates (Fig. 1).

**Figure 2:**
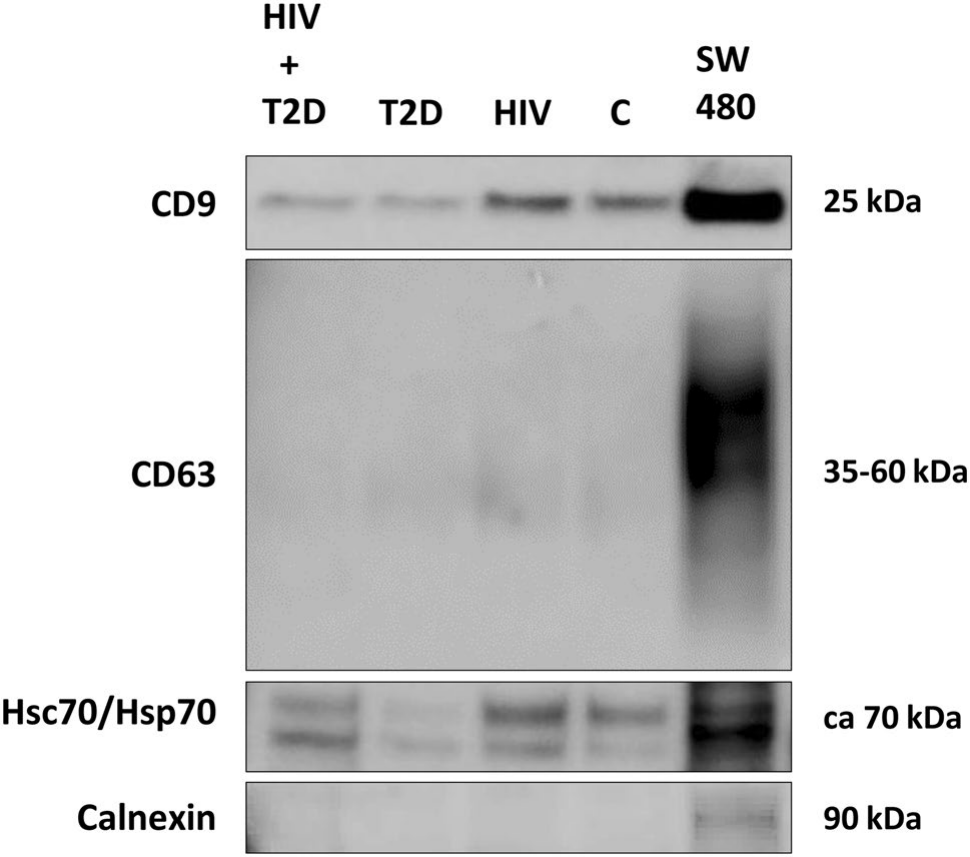
Western blot detection of CD9, Hsc70/Hsp70 and Calnexin in plasma pool EVs. As a positive control, lysate from the colorectal cancer cell line SW480 was used. Full-length blots are presented in Supplementary Fig. 3 (Supplementary Information).

Western blot analysis confirmed the presence of the EV-associated markers CD9 and Hsc70/Hsp70 in the EV preparation of all four plasma pools as well as in the SW480 cell lysate (positive control) (Fig. 2). As expected, the endoplasmic reticulum marker Calnexin (non-EV-associated) was detected in the SW480 cell lysate, but not in the EV isolates.

### HIV-infected individuals with T2D have higher concentration of EVs

**Figure 3:**
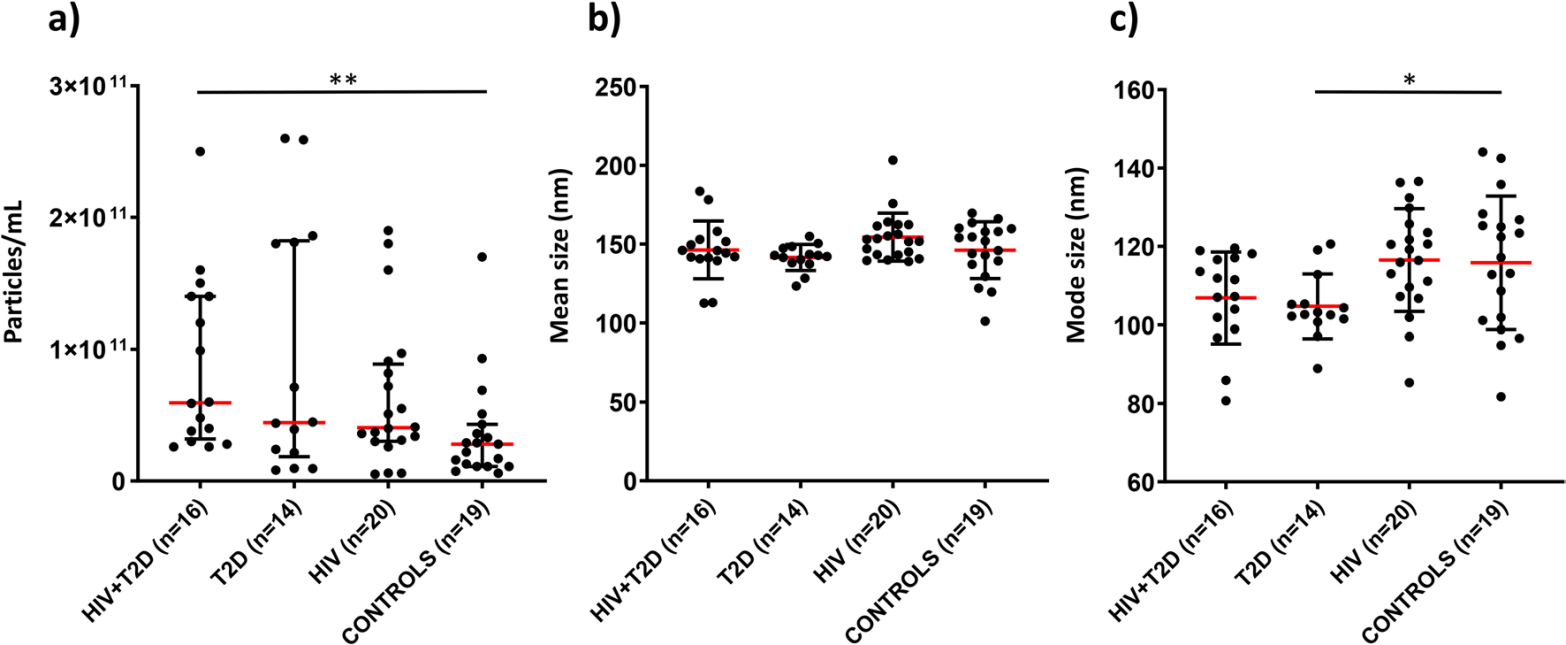
Characterization of EV isolates by NTA. Data are shown as median values with interquartile range (**a**) or mean values with SD (**b**, **c**). All groups were compared using either Mann–Whitney U test (**a**) or parametric t-test (**b**, **c**). *p < 0.05, **p < 0.005.

Individual EV isolates were characterized by nanoparticle tracking analysis (NTA). HIV-infected individuals with T2D had significantly higher particle concentration (hereafter referred to as ‘EV concentration’) than controls, *p* = 0.002 (Fig. 3a). We also observed significantly smaller mode (peak) particle size in type 2 diabetes, and numerically smaller in HIV + T2D, possibly due to higher concentration of small-size exosomes (Fig. 3c). However, there were no significant group differences for mean particle size (Fig. 3b).

### EV concentration correlates with plasma LPS, lipoprotein content and Framingham score, but not with gut microbiota alterations

**Figure 4:**
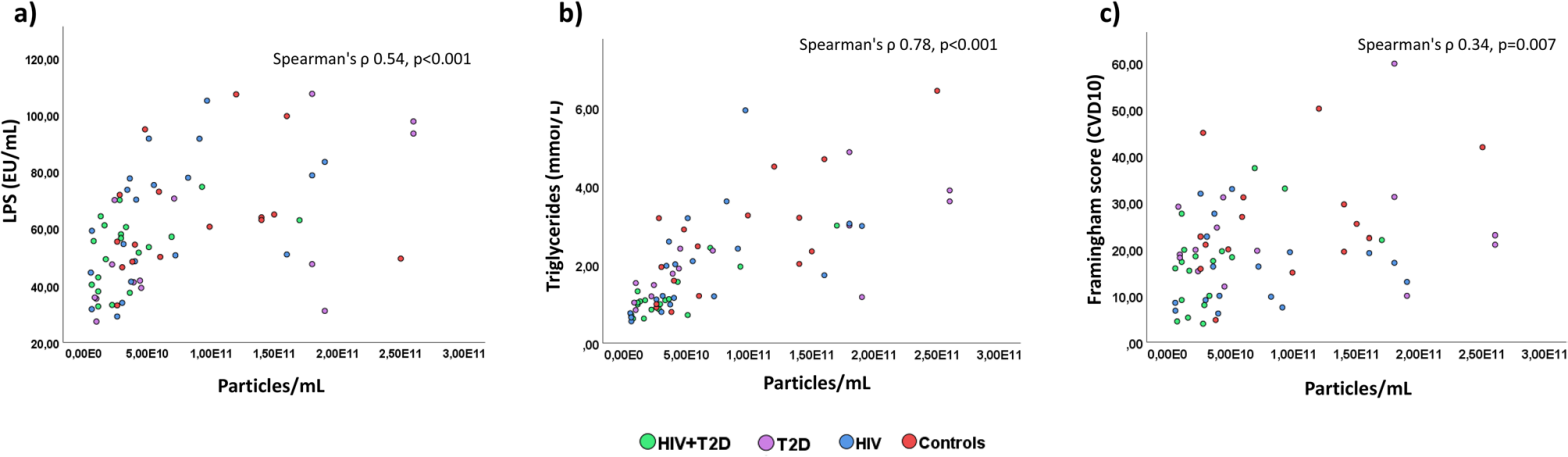
Grouped scatterplots of plasma LPS (**a**), plasma triglycerides (**b**) and Framingham risk score (CVD10) (**c**) by particle concentration as listed in Table 1.

In the overall study cohort, plasma EV concentration correlated with plasma levels of LPS (Fig. 4a) (Table 1), which seemed to be mainly driven by HIV infection (Spearman’s ρ 0.533, *p* = 0.001). From the present cohort, Hoel et al. previously reported reduced microbial alpha diversity in HIV-infected individuals with T2D compared with controls^23^. In our study, plasma EV concentration did not correlate with microbial alpha diversity (Table 1), nor with compositional gut microbiota alterations, such as increased abundance of Enterobacteriales, as previously reported from this cohort^23^.

**Table 1:** Spearman correlation analyses.

	Spearman’s ρ	*p*
**EV concentration (particles/mL) *vs***		
Shannon diversity index	− 0.08	0.50
Observed species	− 0.01	0.94
Enterobacteriales	− 0.07	0.58
Plasma LPS	0.54	< 0.001
Plasma triglycerides	0.78	< 0.001
Plasma cholesterol	0.06	0.60
HDL-cholesterol	− 0.44	< 0.001
LDL-cholesterol	− 0.06	0.61
Framingham score CVD10	0.34	0.007
**Plasma LPS *vs***		
Plasma triglycerides	0.66	< 0.001
Plasma cholesterol	0.30	0.011
HDL-cholesterol	− 0.31	0.008
LDL-cholesterol	0.09	0.47
Framingham score CVD10	0.17	0.16

Plasma lipoprotein particles are transport vehicles for lipids, including triglycerides and cholesterol. Due to their similar size, some lipoprotein particles could be co-isolated with EVs by SEC. We therefore wanted to explore the associations between particle concentration, LPS and lipids, including triglycerides and cholesterol (total, HDL-bound and LDL-bound). Accordingly, EV concentration strongly correlated with plasma triglycerides and negatively with plasma HDL-bound cholesterol (Table 1). We also found that plasma LPS correlated strongly with triglycerides, but also moderately with total cholesterol and negatively with HDL-bound cholesterol (Table 1). There were no associations found for total cholesterol or LDL-bound cholesterol regarding EV concentration. To address the approximate lipoprotein content of our EV isolates, we quantified levels of the commonly abundant Apolipoprotein A1 (ApoA1), a major constituent of HDL particles, and Apolipoprotein B (ApoB), a major constituent of chylomicrons and LDL particles, in EV fractions from pooled plasma of the four study groups. The fractions contained 20–40 ng/mL ApoA1 and 3500–5500 ng/mL ApoB (Supplementary Fig. 1 and Supplementary Methods).

We next aimed to explore EV concentrations and LPS as potential biomarkers related to cardiovascular risk profile as assessed by Framingham risk score. Whereas EV levels correlated significantly with increased Framingham score (Fig. 4), plasma LPS did not. Despite the higher proportion of smokers in the HIV + T2D group (50–100% higher than the other groups), we did not find any difference in EV concentration when comparing smokers (n = 55) to non-smokers (n = 14) in the total study cohort (*p* = 0.7).

### Proteomic analyses related to cardiometabolic disease genes and activated inflammation in EV fractions from study participants

**Figure 5:**
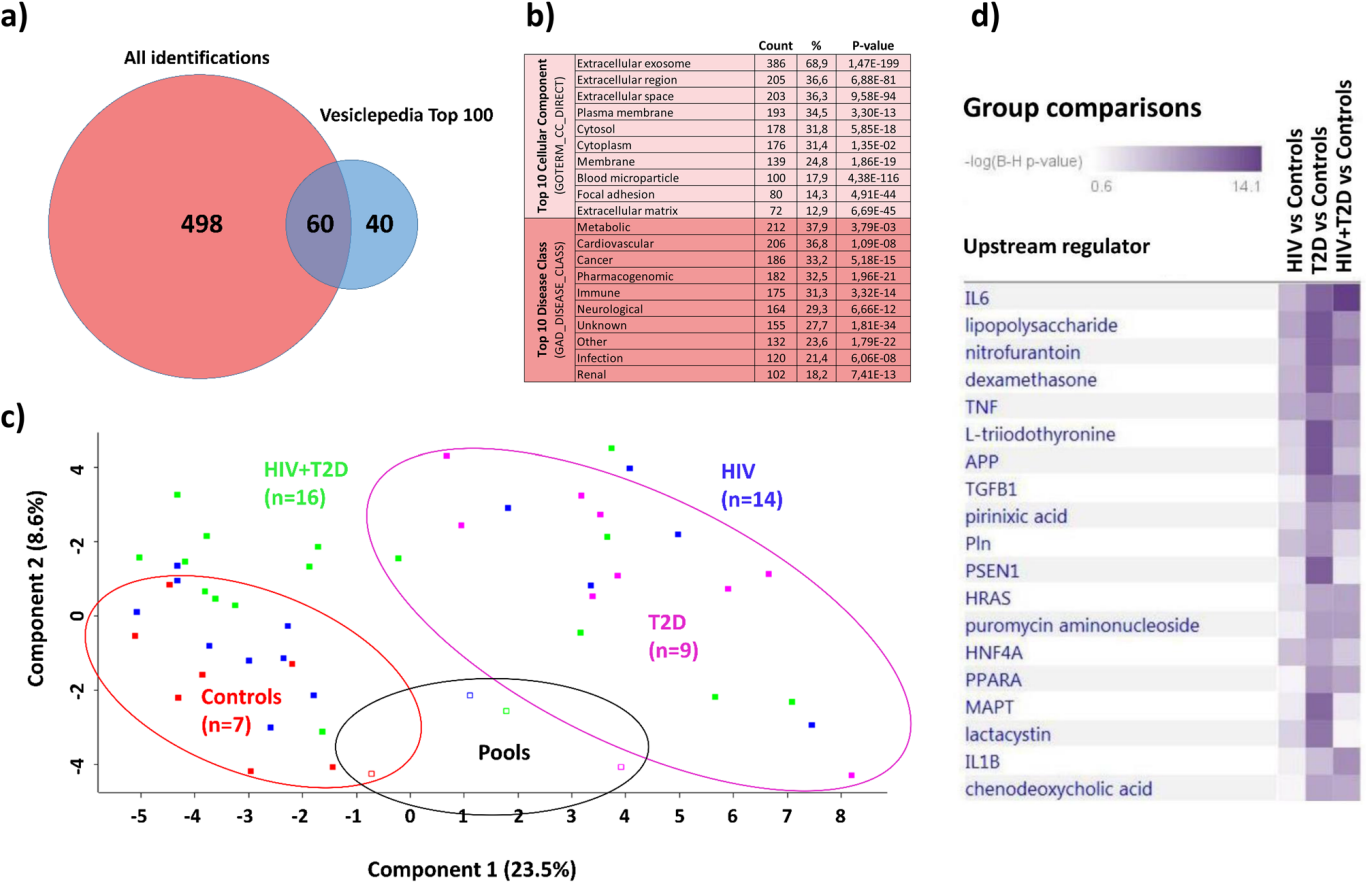
(**a**) Venn diagram showing identified proteins (Swissprot, human) compared with the top 100 identified EV-associated proteins from Vesiclepedia database. (**b**) Selected gene ontology analyses of the identified proteins sorted by gene count. (**c**) Principal component analysis of all individual samples analyzed by MS, HIV + T2D in green (n = 16), T2D only in purple (n = 9), HIV only in blue (n = 14) and controls in red (n = 7). Open squares are patient group pools. (**d**) Ingenuity Pathway Analysis showing the top upstream regulators for the differentially abundant proteins in the sample groups (Supplementary File 1).

To further elucidate on the possible link between EVs, microbial translocation and cardiovascular risk, we characterized the protein content of EVs using quantitative, label-free mass spectrometry (MS)-based proteomics. In total, we identified 558 human proteins in the EV isolates (Supplementary File 1). From the identified proteins, 60 matched to the Vesiclepedia Top 100 EV protein database (Fig. 5a), including the tetraspanins CD9, CD81 as well as Flotillin-1, often reported as EV-associated markers. In addition, several common high-abundant plasma proteins were among the top 50 identified proteins according to MS-intensity values, such as apolipoproteins, albumin, fibrinogen, immunoglobulins and cytoskeleton-associated proteins (Supplementary File 1). However, of the top 50 most abundant proteins, we also detected CD5 antigen-like (CD5L) and galectin-3-binding protein (LGALS3BP), previously suggested as suitable markers of plasma-derived EVs in proteomic identification by MS^24,25^.

Gene ontology analyses of the whole dataset showed that the top cellular compartments of matched protein identifications largely related to extracellular vesicles and extracellular region (Fig. 5b). Metabolic disease and cardiovascular disease were identified as the top disease classes (Fig. 5b). Furthermore, database search against bacterial proteins resulted in 30 bacterial protein hits originating from 27 different species (counting the first detected species listed by MaxQuant), of which 19 belonged to the bacterial phylum Proteobacteria (Supplementary file 2).

To compare the proteomic fingerprint of EVs in the different study groups, we first evaluated the samples by principal component analysis (PCA) (Fig. 5c). Samples from the HIV + T2D as well as the HIV only group, were similarly distributed across the principal components, whereas samples from the T2D only group and the controls seemed to cluster separately. EV isolates from plasma pools distributed representatively according to the groups, except for the T2D pool, positioned slightly outside the clustering of the individual samples.

We then compared proteins in the different patient groups against controls (t-test, *p* < 0.05) which showed 50, 111 and 26 differentially abundant proteins in HIV + T2D, T2D and HIV groups compared to controls, respectively (Supplementary File 1^1^. Pathway analysis showed that in HIV-infected individuals with T2D, proteins were largely related to upstream regulators of inflammatory pathways, including Interleukin-6 (IL-6), as well as inflammasome activation by IL-1β (Fig. 5d). Alterations were also apparent in upstream regulation of LPS in all group comparisons, particularly in T2D compared with controls. In addition, TGF-β, involved in immune regulation, was identified as an upstream regulator, particularly in T2D irrespective of HIV status. Finally, the abundance of the identified plasma EV markers CD5L and LGALS3BP were higher in HIV + T2D than controls, and CD5L higher in T2D than controls (Supplementary file 1).

### In vitro pro-inflammatory effect of plasma EVs from patients with HIV and type 2 diabetes

To investigate a possible contribution of circulating EVs to low-grade inflammation in patients with HIV and type 2 diabetes, we stimulated human primary monocytes with EVs from patients and controls of the present cohort. Patient EVs seemed to trigger a slightly higher pro-inflammatory response in the monocytes than EVs from controls, as measured by release of IL-1β and IL-6 to the supernatant after three hours of incubation (Supplementary Fig. 2a and Supplementary Methods).

While we hypothesize that the number of circulating bacterial EVs in patients with HIV and type 2 diabetes could be key contributors to this pro-inflammatory effect, and presumably higher in these individuals than in healthy ones, the exact ratio of bacterial to host EVs remains unknown. We therefore also stimulated monocytes with bacterial OMVs from *Neisseria meningitidis* in different concentrations, finding a dose-dependent response of IL-1β and IL-6 in the monocytes (Supplementary Fig. 2b).

## Discussion

In the present study, we aimed to explore the potential role of plasma EVs as mediators of microbial translocation and drivers of cardiometabolic risk. We observed higher EV concentrations in patients with combined HIV and T2D compared with controls, and EV levels correlated closely with plasma levels of LPS and triglycerides, but not with gut microbiota alterations. Furthermore, EV concentration but not plasma LPS was associated with an increased 10-year risk of cardiovascular disease. Lastly, the proteomic content of plasma EVs isolated from HIV-infected individuals with T2D, was at top related to metabolic and cardiovascular disease genes and upstream regulation of inflammatory pathways, including IL-6 and inflammasome activation (IL-1β). Proteins of bacterial origin were also identified, mostly from lipopolysaccharide-producing Proteobacteria.

Isolation of EVs from plasma samples is challenging due to the large amount of abundant proteins, including lipoproteins. In recent years, size exclusion chromatography (SEC) has been utilized as a fast and gentle stand-alone method for isolation of biologically active EVs from human plasma^24,26,27^. While SEC does not completely separate plasma EVs from lipoprotein particles, several combined approaches have been suggested to further improve the purity of EV-enriched fractions from plasma, including the combination of liquid chromatography with density gradient separation^28–30^. However, the approaches seem to be at the expense of EV yield, and often require larger sample volume than what is available from clinical material. Furthermore, there are emerging results of interactions of EVs with other components of the plasma, including proteins and lipoproteins, and their possible significance to both biological functions and biomarker discovery^31,32^. In our study, we have taken into account that the EV isolates contain a fraction of similarly sized particles to EVs, such as lipoprotein particles, which might explain some of our findings.

Increased plasma EV levels have been reported in several disease states, including HIV^33–35^ and T2D^36,37^. In our study, we observed significantly higher EV concentrations in individuals with HIV and T2D than controls, and numerically higher in HIV only and T2D only compared to controls. The non-significant differences between these disease groups might be due to the relatively low sample size in our study. Of note, the proportion of smokers in our study was around 50–100% higher in the HIV + T2D group compared to other groups (Table 2), hence this might influence our findings. A possible link between smoking and HIV pathogenesis has also previously been described^38,39^. Nevertheless, we did not find any differences in EV concentration regarding smoking. Hence, our results support a potentially additive effect of HIV infection and T2D on EV concentration in the circulation.

**Table 2:** Characteristics of the study participants.

	HIV + T2D	T2D	HIV	Controls	*p*
N	16	14	21	20	
Age (years)	57 (53–62)	58 (54–61)	55 (51–58)	58 (55–61)	0.55
Gender (% male)	88	71	95	90	0.25
Smoker (%)	31	14	14	20	0.64
Use of medication (%)					
PI	38	–	67	–	0.10
NNRTI	63	–	29	–	0.052
Statins	69	71	5	15	< 0.001
Betablockers	25	7	5	0	0.051
ACE inhibitor/ATII Antagonist	56	36	10	25	0.019
Oral antidiabetics	81	71	–	–	0.68
Insulin	25	14	–	–	0.66
Physical activity (< 1/1–2/ ≥ 3 times/week)	44/31/19	29/21/29	33/29/33	20/35/45	0.47
HIV transmission (MSM, heterosexual, IDU) (%)	69/6/13	–	71/19/0	–	0.38
Time on stable ART (months)	132 (91–174)	–	122 (82–161)	–	0.670
HIV RNA (copies/mL)	29 (14–43)	–	30 (18–42)	–	0.62
CD4 count (cells/µL)	630 (488–773)	1088 (901–1274)^c,d^	580 (460–699)	870 (704–1036)^b,c,d^	< 0.001
LPS (EU/mL)	65 (54–76)	56 (41–72)	62 (52–72)	60 (43–77)	0.56
Total cholesterol (mmol/L)	4.6 (4.2–4.9)	4.4 (3.8–4.9)	5.5 (5.1–6.0)^b,d^	5.3 (4.9–5.8)^b,d^	0.001
HDL cholesterol (mmol/L)	1.2 (1.0–1.4)	1.3 (1.1–1.5)	1.4 (1.2–1.6)	1.7 (1.4–1.9)^b,d^	0.009
LDL cholesterol (mmol/L)	2.4 (2.0–2.9)	2.4 (1.9–3.0)	3.5 (2.0–3.9)^b,d^	3.4 (3.1–3.7)^b,d^	< 0.001
Triglycerides (mmol/L)	2.7 (1.8–3.5)	2.2 (1.5–2.9)	1.9 (1.3–2.5)	1.3 (1.0–1.6)^b,d^	0.008
Fasting BG (mmol/L)	8.0 (6.6–9.4)	8.8 (7.5–10.1)	5.3 (5.1–5.5)^b,d^	5.2 (5.0–5.5)^b,d^	< 0.001
HbA1c (mmol/mol)	48 (43–53)	57 (50–63)	35 (33–37)^b,d^	37 (36–38)^b,d^	< 0.001
Systolic BP (mmHg)	129 (122–136)	137 (131–144)	129 (122–136)	136 (130–142)	0.13
Diastolic BP (mmHg)	82 (77–87)	87 (81–92)	80 (76–84)	84 (80–87)	0.23
BMI (kg/m^2^)	27 (25–29)	28 (26–30)	25 (23–27)	25 (24–26)^b^	0.045
Framingham risk score (CVD10)	26 (19–33)	24 (17–31)	15 (11–20)^b,d^	17 (13–21)^b,d^	0.011

*p* value refers to one-way ANOVA for continuous data and Chi-Square or Fisher’s exact test for categorical data. Results are given as % or mean and 95% CI.

*PI* Protease inhibitor, *NNRTI* Non-nucleoside reverse-transcriptase inhibitor, *ACE* Angiotensin-converting enzyme, *ATII* Angiotensin II, *MSM* Men who have sex with men, *IDU* Intravenous drug use, *ART* Antiretroviral therapy, *LDL* Low-density lipoprotein, *HDL* High-density lipoprotein, *BMI* Body mass index, *BP* Blood pressure.

^b,c,d^ refers to t-test; ^b^*p* < 0.05 versus T2D, ^c^*p* < 0.05 versus HIV, ^d^*p* < 0.05 versus HIV + T2D.

We observed an inter-correlation between EV concentration and plasma levels of both LPS and triglycerides, in line with others reporting on associations between LPS and triglycerides^12^. Thus, the question arises whether circulating LPS is translocated in complex with triglyceride-rich lipoprotein particles, such as chylomicrons, or with bacterially derived EVs, or even as soluble molecules. Of note, 90% of plasma LPS has been shown to be lipoprotein-bound, with highest affinity for HDL, medium affinity for low-density lipoprotein (LDL), and low affinity for very LDL (VLDL), due to the differential phospholipid composition^40,41^. While EVs separated from healthy plasma incubated with bacterial OMVs seem to co-localize with the majority of LPS, but not with HDL particles, following SEC fractionation (data not shown), the exact translocation route of vesicle-associated LPS remains unclear. Nevertheless, our findings suggest that there is a potential interplay between EVs and lipoprotein particles involved in the process of microbial translocation.

We have recently reported that HIV-associated gut microbiota alterations, including higher relative abundance of gram-negative LPS-producing Gammaproteobacteria, are closely associated with abdominal obesity and excess metabolic risk^42^. Although individuals with combined HIV and T2D in the present cohort had increased abundance of gram-negative Enterobacteriales^23^, we found no correlation between compositional gut microbiota alterations and circulating EVs. While the observed gut microbiota alterations does not seem to drive the association of increased EV concentration or plasma levels of LPS, alterations in other taxa of LPS-producers, in particular mucosa adherent microbes not captured in regular stool samples, could still lead to increased levels of LPS-positive OMVs in the circulation. While gut microbe-derived EVs have been shown to translocate across the intestinal barrier and distribute into the insulin-responsive tissues and induce insulin resistance and glucose intolerance^43^, we detected several bacterial proteins in the EV isolates, of which approximately 70% of the identified species origins belonged to the bacterial phylum Proteobacteria, which are LPS-producing gram-negative bacteria. Although the taxonomic classification of bacterial proteins by mass spectrometry in our study was suboptimal due to the low numbers of identified peptides, the findings might be supportive of bacterial translocation beyond LPS in the circulation.

In our study, EV concentration, but not LPS, correlated with increased 10-year Framingham risk score for cardiovascular disease. This correlation could partly be affected by increased plasma triglycerides, although triglycerides is not involved in the Framingham score, in contrast to cholesterol, which did not correlate with EV concentration. To further explore the role of EVs as potential drivers of cardiovascular risk beyond lipoproteins using proteomics, we identified 50, 111 and 26 differentially abundant proteins in HIV + T2D, T2D and HIV groups compared to controls, respectively. Importantly, we identified proteins involved in upstream regulation of inflammatory pathways, most prominently the upstream regulator of IL-6. Moreover, our in vitro experiments support a possible contribution of plasma EVs to the low-grade inflammation observed in the patient groups, as judged by the slightly higher production of IL-1β and IL-6 in the EV-exposed monocytes. Of note, the presence of circulating LPS-positive bacterial EVs that are able to induce immune activation has been reported in plasma in patients diagnosed with IBD, HIV and cancer therapy-induced intestinal mucositis^14^. Hence, we speculate that translocated bacterial EVs from the gut to the circulation could contribute to the observed low-grade inflammation in our study, supported by a dose-dependent pro-inflammatory response in monocytes treated with OMVs from *Neisseria Meningitidis*. Another possible contribution to chronic inflammation may be EVs released from latently HIV-infected cells that transfer viral proteins and pro-inflammatory molecules to circulating immune cells, even in the presence of stable ART^44^. This could also be the case for the HIV-infected individuals in our study; however this could not explain the presence of pro-inflammatory EVs in individuals with T2D.

Increased levels of pro-inflammatory IL-6 have in several publications been a marker for cardiovascular events in PLWH and in the general population^45–47^. Moreover, upstream regulation of inflammasome activation and IL-1β could be relevant as we recently reported IL-1 activation as a predictor of first-time myocardial infarction in PLWH^48^. Of note, IL-6 and IL-1 are both available for pharmaceutical intervention, and in the large CANTOS trial, IL-1 inhibition decreased the risk of recurrent myocardial infarction^49^. The observed alterations in upstream regulation of LPS in all patient groups, particularly in T2D, could possibly be due to the high number of identified proteins in this group.

Furthermore, we observed higher levels of novel plasma EV markers CD5L and LGALS3BP in individuals with HIV and T2D than controls. CD5L and LGALS3BP are secretory proteins belonging to the superfamily of scavenger receptor cysteine-rich (SRCR) proteins, mostly expressed by macrophages in lymphoid and inflamed tissues^50,51^. During the last decade, CD5L has gained increasing importance as a critical player in pattern-recognition of microbial components, as well as in controlling key mechanisms in inflammatory responses related to infection, atherosclerosis and cancer^51^. Moreover, high LGALS3BP levels has been associated with long-term mortality in in coronary artery disease^52^.

A limitation of the present study is the cross-sectional design and the relatively small number of study participants, with several comorbidities and treatment therapies, excluding the possibility to draw causal conclusions. Moreover, the available plasma volume in many clinical studies is insufficient to efficiently separate EVs from abundant plasma proteins, potentially obscuring less abundant EV-associated proteins. This also applies to the present study, although the proteomics analyses suggest that EV content could be involved in central inflammatory pathways and mediate cardiovascular risk beyond that of lipoproteins. The obvious strengths of the present study include the possibility to compare patient groups with different chronic diseases, and to compare EV-related measures with a wide range of clinical and circulating markers as well as microbiota profiles.

In conclusion, our exploratory results suggest a potential interplay between EVs and lipoprotein particles involved in the process of microbial translocation in individuals with HIV and T2D. The proteomic content suggests a potential contributing role of EVs in chronic inflammation and cardiovascular risk development. However, future studies of plasma EVs from clinical cohorts will depend on larger sample sizes and improved innovative methodology to separate similarly sized particles in low-volume clinical samples, to confidently increase our understanding of circulating EVs in the process of microbial translocation and increased cardiometabolic risk.

## Methods

### Study participants and baseline characteristics

In the present study, 71 individuals were included from a Danish cross-sectional cohort with EDTA plasma and stool samples available^53^; 16 individuals with concomitant HIV and type 2 diabetes (HIV + T2D), 14 with type 2 diabetes only (T2D), 21 with HIV only (HIV) and 20 healthy controls. All HIV-infected individuals were included at Department of Infectious Diseases, University Hospital of Copenhagen, Rigshospitalet and Hvidovre Hospital. Individuals with T2D only were included from Department of Endocrinology, and Center of Inflammation and Metabolism, University Hospital of Copenhagen, Rigshospitalet. Healthy controls were included from the hospital staff. The study population characteristics are displayed in Table 2. All individuals with HIV were on stable antiretroviral therapy (ART) and virally suppressed, whereas all individuals with T2D were treated with diet and/or oral antidiabetics and/or insulin as previously reported^53^.

Framingham Risk Score was calculated as previously described^53^, using age, gender, weight, height, smoking status, diabetes, left ventricular hypertrophy on electrocardiogram, systolic blood pressure, total cholesterol and HDL-cholesterol.

### Sample collection and processing

Fasting EDTA whole blood was placed immediately on ice and centrifuged at 2500 g for 15 min at 4 °C, within 60 min after collection. Plasma was stored at − 80 °C. At inclusion, study participants were instructed to collect stool samples in containers without preservatives and shipped by mail to Rigshospitalet, where it was stored at − 80 °C.

### Blood biochemistry analyses

Glucose, HbA1c, lipid parameters, CD4 T cell count and HIV RNA were measured as routine analyses. Lipopolysaccharide bioactivity was analyzed using the limulus amebocyte lysate (LAL) colorimetric assay (Lonza, Walkersville, MD) following the manufacturer’s recommendations and performed under regular assessment of possible contamination of tubes and equipment used for collection, processing and analysis. Pyrogen-free tubes were used throughout, samples were diluted tenfold to avoid interference with background color, vortexed and preheated to 68 °C for 10 min prior to analyses to dissolve immune complexes as previously described^54^. All samples were analyzed in duplicates.

### Gut microbiota analyses

We have previously described gut microbiota analyses in detail from the present cohort^23^. In brief, the 16 s rRNA gene was amplified by PCR from fecal DNA extracts using universal primers targeting the V3–V4 region along with TruSeq Ilumina adapters. Pooled and normalized amplicons were purified and eluted for sequencing on the Illumina MiSeq platform using 250 base pair paired-end protocol at the Norwegian Sequencing Centre (Oslo, Norway). For bioinformatic analyses, paired-end reads were pre-processed and analyzed using QIIME as previously described^23^.

### Isolation and characterization of plasma EVs

#### Size exclusion chromatography

Plasma EVs from individual samples were isolated using qEV size exclusion chromatography columns (IZON Science, Oxford, UK) according to the manufacturer’s protocol. Briefly, columns were equilibrated with 20 mL of 0.1 µm-filtered phosphate buffered saline (PBS) (Gibco, Life Technologies) before 400 µL of plasma was added and 500 µL fractions were collected. Fractions 7–9, containing the majority of EVs, were pooled and concentrated using Amicon Ultra-2 10 kDa Centrifugal Filter Devices (Merck Millipore). Similarly, for pooled plasma EV preparations of each patient group, a total of 1.4 mL plasma were pooled from 60 to 100 µL plasma per individual (HIV + T2D: n = 21, T2D: n = 14, HIV: n = 23, Controls: n = 23) and isolated in three runs on the qEV column. All samples were stored at − 80 °C.

#### Transmission electron microscopy

Electron microscopy samples were prepared essentially as described^55^. After loading EVs from group pools onto 200 mesh copper grids, the samples were fixed with 2% paraformaldehyde in 0.1 M NaPO4 buffer, pH 7.0 (NaPO4). Then, grids were washed with the NaPO4 buffer and deionized water, negatively stained with 2% neutral uranyl acetate and embedded in methyl cellulose uranyl acetate mixture (1.8/0.4%). All samples were viewed using a TECNAI 12 transmission electron microscope (FEI, Hillsboro, OR, USA) operating at 80 kV. Images were taken with a Veleta 2kX2k CCD camera and processed with iTEM software (Olympus Soft Imaging Solutions).

#### Western blot

Twenty µL of EVs from group pools were added 6 µL RIPA 5 × buffer (Thermo Fisher Scientific) and 1.25 µL protease inhibitor (cOmplete, Mini, EDTA-free Protease Inhibitor Cocktail 25 × , Roche), sonicated for 20 s and lysed on ice for 15 min. Then, 10 µL LDS Sample Buffer (Invitrogen) was added. Hsc70/Hsp70 and calnexin was analyzed under reducing conditions and received 4 µL Bolt Sample Reducing Agent, whereas CD9 was added 4 µL of PBS before all lysates were heated for 10 min at 70 °C. Proteins were separated on Bolt 4–12% Bis–Tris Plus Gels with Bolt MES SDS Running Buffer (both Invitrogen) and transferred to 0.2 µm PVDF Blotting Membranes (Invitrogen). Membranes were blocked with 1% casein (Western Blocking Reagent, Sigma Aldrich) in tris-buffered saline with 0.1% Tween 20 (TBS-T) for 1 h at room temperature (RT), then incubated with primary mouse monoclonal antibodies (anti-CD9, Invitrogen, 10626D (Ts9), 1:750; anti-CD63, Invitrogen, 10628D (Ts63), 1:500; anti-Hsc70/Hsp70, Enzo Life Science, ADI-SPA-820, 1:1000; anti-Calnexin, Invitrogen, MA5-15389, 1:1000) overnight at 4 °C. The membranes were then washed three times 20 min with TBS-T before incubation with horseradish peroxidase-coupled secondary antibody (Mouse TrueBlot Ultra, Rockland Immunochemicals, Pottstown, USA) for one hour at RT. Following triplicate washing with TBS-T, blots were imaged using SuperSignal West Dura Extended Duration Substrate (Thermo Scientific) and Amersham Imager 600 (GE Healthcare, UK). Original images of full-length membrane blots can be found in Supplementary Information (Supplementary Fig. 3).

#### Nanoparticle tracking analysis

EV isolates were analyzed by nanoparticle tracking analysis as previously described^56^. Briefly, EVs were diluted in 0.02 µm-filtered PBS (Whatman Anotop™25, GE Healthcare Life Science, Buckinghamshire, UK), to reach the measurement range (1.0–9.0 × 10^8^ particles/mL). Samples were loaded into a NanoSight NS500 instrument (Malvern, Amesbury, UK) equipped with a sCMOS camera, using a syringe pump and flow speed 20. For each sample, three 60-s videos were captured, typically at camera level 14 and detection threshold 3, then analyzed using NTA 3.1 software Build 3.1.54.

#### Proteomics

50 µL of EVs were mixed with 30 µL ProteaseMAX™ Surfactant (Promega) in 50 mM NH4HCO3. The proteins were reduced, alkylated and digested into peptides with trypsin (Promega) according to the ProteaseMax protocol. The resulting peptides were desalted and concentrated with EvoTips (EvoSep). Each peptide mixture was analyzed by EvoSepOne coupled to QExactive HF (ThermoElectron, Bremen, Germany) with 15 cm EvoSep column using a 30 samples/day method. The resulting MS raw files were submitted to the MaxQuant software version 1.6.1.0 for protein identification and label free quantification. Carbamidomethyl (C) was set as a fixed modification and acetyl (protein N-term), carbamyl (N-term) and oxidation (M) were set as variable modifications. First search peptide tolerance of 20 ppm and main search error 4.5 ppm were applied. Trypsin without proline restriction enzyme option was used, allowing two miscleavages. The number of minimal unique + razor peptides was set to 1, and the allowed FDR was 0.01 (1%) for peptide and protein identification. Label-free quantitation was employed with default settings. The Uniprot databases with ‘human’ entries (September 2018) as well as ‘bacteria’ entries (May 2020) were used for the database searches. Perseus software version 1.6.1.3 was used for the statistical analysis of the results. Known contaminants as provided by MaxQuant and identified in the samples were excluded from further analysis. Proteomic venn diagram was created using Funrich tool version 3.1.3 (www.funrich.org). Gene ontology analyses of identified proteins were performed using David functional annotation tool (https://david.ncifcrf.gov), and group comparisons of proteomic data were done using Ingenuity® Pathway Analysis (IPA; QIAGEN inc., https://www.qiagenbioinformatics.com).

#### Statistical analysis

Normally distributed continuous data were analysed using parametric two-sample T-test for comparison of two groups or one-way ANOVA for multiple groups. Skewed data were log-transformed and analysed by parametric statistics or analysed using non-parametric Mann–Whitney U Test or Kruskal–Wallis for comparison between two or more groups, respectively. Categorical data was analysed using Pearson chi-square test. Fisher’s exact test was used whenever applicable. Correlations were performed by Spearman correlation. Two-tailed *p* values < 0.05 were considered significant. Statistical analyses were performed using SPSS version 26 (SPSS, Inc.; Chicago, IL, USA) and GraphPad Prism 8.3.0 (GraphPad Software, San Diego, CA, USA). For proteomic data, group comparisons were performed by two-sample T-tests (*p* < 0.05) on Log10-transformed data using Perseus software version 1.6.1.3, requiring at least 50% valid values in at least one group. Missing values were imputed from normal distribution.

### Ethical considerations

The study was performed in accordance with the Helsinki declaration, approved by the The Regional Committees on Health Research Ethics for Southern Denmark (VEK no. H-4-2012-076 and H-19028857) and the Danish data protection agency, as well as the Regional Committees for Medical and Health Research Ethics in Norway (REK no. 31412). Written informed consent was obtained from all individuals.

## Supplementary Information

Supplementary Information 1.

Supplementary Information 2.

Supplementary Information 3.

## Data Availability

The datasets generated and/or analyzed during the present study beyond the supplementary anonymized raw data are not publicly available due to Danish and Norwegian legislation about general data protection regulation, but data are available from the corresponding author on reasonable request. Written details on experimental procedures have been submitted to the EV-TRACK knowledgebase (EV-TRACK ID: EV200050)^57^.
