# Supplementary material for: Plasma extracellular vesicles in people living with HIV and type 2 diabetes are related to microbial translocation and cardiovascular risk
Source: Sci Rep. 2021 Nov 9;11:21936. doi: 10.1038/s41598-021-01334-y (PMC8578564; doi:10.1038/s41598-021-01334-y)
Supplement: Supplementary file 3 — Supplementary Information 3. [file 41598_2021_1334_MOESM3_ESM.pdf]

Supplementary information

Plasma extracellular vesicles in people living with HIV and type 2 diabetes are related to microbial translocation and cardiovascular risk

Beate Vestad*, MSc[1,2,3], Tuula A. Nyman†, PhD[4], Malene Hove-Skovsgaard†, MD[5], Maria Stensland, PhD[4], Hedda Hoel, MD[1,2,6], Anne-Marie Siebke Trøseid, BSc[3,7], Trude Aspelin, PhD[3,7], Hans Christian D. Aass, PhD[3,7], Maija Puhka, PhD[8], Johannes R. Hov, MD, PhD[1,2,9], Susanne Dam Nielsen MD, PhD, DMsc [5], Reidun Øvstebø††, PhD[2,3,7], Marius Trøseid††, MD, PhD[1,2,10]

[1]Research Institute of Internal Medicine, Oslo University Hospital Rikshospitalet, Oslo, Norway, [2]Institute of Clinical Medicine, University of Oslo, Oslo, Norway, [3]Norwegian Society for Extracellular Vesicles, NOR-EV, Norway, [4]Department of Immunology, Institute of Clinical Medicine, University of Oslo and Oslo University Hospital Rikshospitalet, Oslo, Norway, [5]Department of Infectious Diseases, University Hospital of Copenhagen Rigshospitalet, Copenhagen, Denmark, [6]Medical Department, Lovisenberg Diaconal Hospital, [7]The Blood Cell Research Group, Department of Medical Biochemistry, Oslo University Hospital, Ullevål, Oslo, Norway, [8] Institute for Molecular Medicine Finland FIMM, EV and HiPrep Cores, University of Helsinki, Helsinki, Finland, [9]Norwegian PSC Research Center and Section of Gastroenterology, Division of Surgery, Inflammatory Medicine and Transplantation, Oslo University Hospital Rikshospitalet, Oslo, Norway, [10]Section of Clinical Immunology and Infectious diseases, Oslo University Hospital Rikshospitalet, Oslo, Norway

†these authors have contributed equally to the manuscript

††these authors have contributed equally to the manuscript

Supplementary file 1: Proteomic data from search against human proteins (Swissprot). Sheet 'proteinGroups' shows the protein identification and label-free quantification data from MaxQuant search, and sheet 'Perseus' shows results from additional data processing and statistical analyses performed in Perseus software. Data are anonymized, and individual samples within each group are equally labeled with group name 1-4, representing Controls, T2D only, HIV only and HIV+T2D, respectively. Sheet 'Filtered IDs vs Vesiclepedia' lists the identified proteins compared with Vesiclepedia database.

Supplementary file 2: Proteomic data from search against bacterial proteins (Uniprot). Sheet 'proteinGroups' shows the protein identification and label-free quantification data from MaxQuant search. Data are anonymized, and individual samples within each group are equally labeled with group name 1-4, representing Controls, T2D only, HIV only and HIV+T2D, respectively. Sheet 'Taxonomy' shows taxonomic classification of origin species from first detected Majority Protein ID of each identification.

Supplementary Figures:

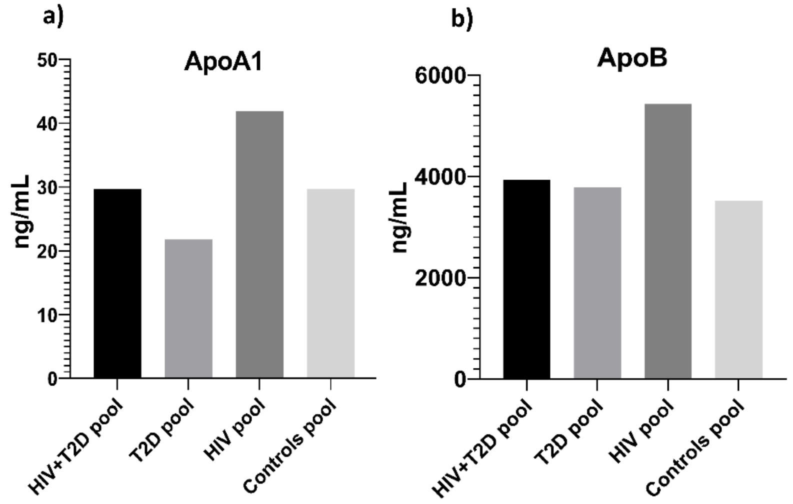

Supplementary Figure 1. Levels of abundant apolipoproteins in unconcentrated EV fractions from pooled plasma.

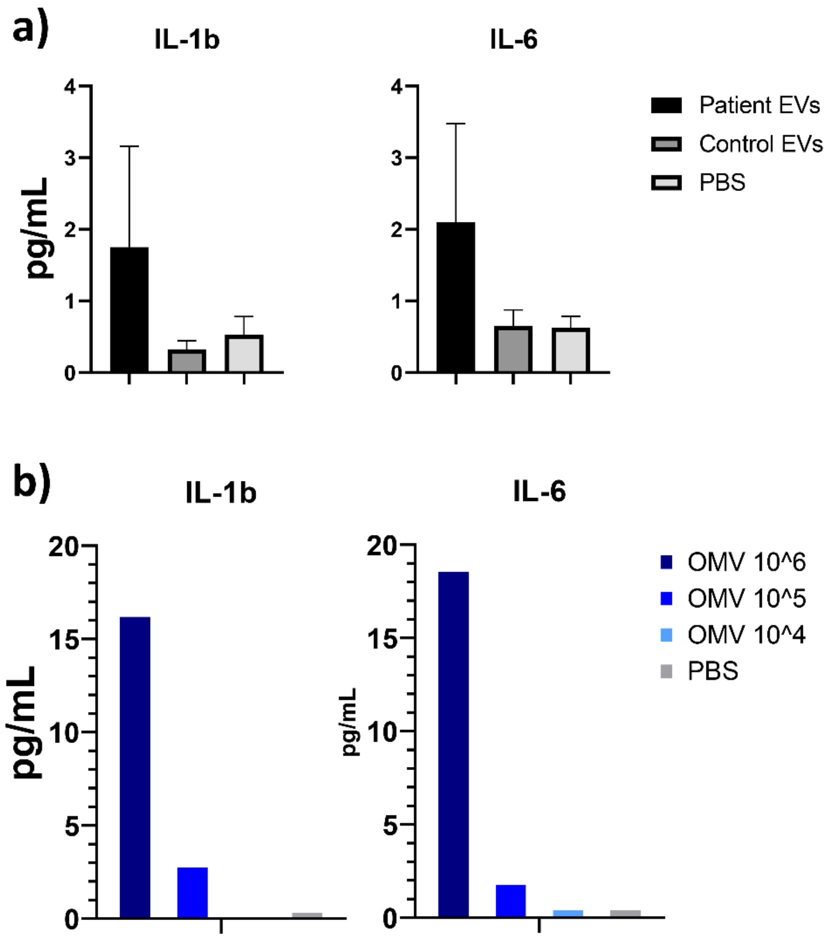

Supplementary Figure 2. Pro-inflammatory effect of plasma EVs from patients with HIV and/or type 2 diabetes compared with controls (a), and bacterial outer membrane vesicles (OMVs) from *Neisseria Meningitidis* (b), on primary human monocytes *in vitro*. Results in (a) are shown as mean values with SD from technical replicates in the same experiment; 9 replicates for patient EVs (3 from each patient group) and 2 replicates for control EVs.

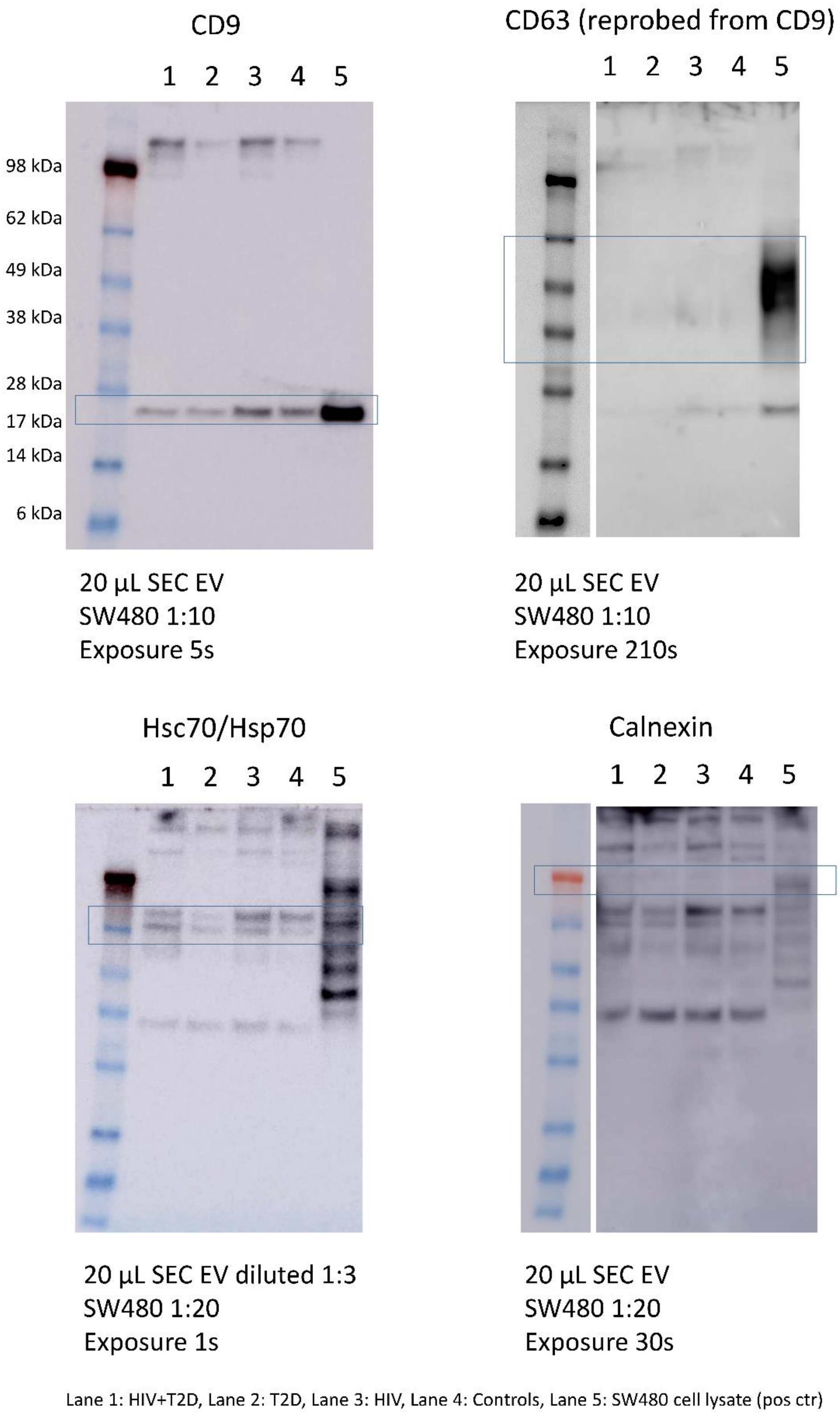

Supplementary Figure 3. Uncropped full-length membrane blots used for Figure 2 (cropped versions are highlighted in blue squares).

Supplementary Methods

Apolipoprotein ELISA assay

Quantification of Apolipoprotein A1 (ApoA1) and Apolipoprotein B (ApoB) in unconcentrated EV isolates from plasma pools was performed using the Human ApoA1 and ApoB Duplex ELISA Kit, Cat no. STA-361 (Cell Biolabs Inc., San Diego, CA, USA) following the manufacturer's protocol, using sample dilution 1:5.

In vitro experiments

Primary human monocytes were isolated from healthy blood by density gradient centrifugation and counter current elutriation as previously described by our group (1) and incubated with EVs. Briefly, 0.15 million cryopreserved monocytes were thawed and dissolved in 100 µL RPMI containing 10% exosome-depleted FBS (Gibco, Thermo Fisher Scientific) and incubated in a 96-well plate with 50 µL EVs from plasma pools of patients with HIV and/or type 2 diabetes or controls (10^8-10^9 particles per well), or with 50 µL OMVs from *Neisseria Meningitidis* (10^4-10^6 per well) for 3 hours at 37 °C, 5% CO_2. Cytokine analysis of cell culture supernatants were performed with a multiplex kit (Bio-rad, Hercules, CA, USA) containing antibody conjugated beads against IL-1β and IL-6. Samples were thawed on ice, spun down at 10 000 xg and diluted (factor 1.56) prior plate loading in duplicate wells. Samples were processed on a Luminex IS 200 instrument (Bio-rad, Hercules, CA, USA).

Supplementary References

1. Lund PK, Joo GB, Westvik AB, Ovstebo R, Kierulf P. Isolation of monocytes from whole blood by density gradient centrifugation and counter-current elutriation followed by cryopreservation: six years' experience. ScandJClinLab Invest. 2000;60(5):357-65.
